# A simple, rapid, and sensitive fluorescence-based method to assess triacylglycerol hydrolase activity

**DOI:** 10.1016/j.jlr.2021.100115

**Published:** 2021-09-09

**Authors:** Sujith Rajan, Hazel C. de Guzman, Thomas Palaia, Ira J. Goldberg, M. Mahmood Hussain

**Affiliations:** 1 Department of Foundations of Medicine, NYU Long Island School of Medicine, and Diabetes and Obesity Research Center, NYU Langone Hospitals - Long Island, Mineola, NY, USA; 2 Department of Environmental Medicine, NYU Grossman School of Medicine, New York, NY, USA; 3 Division of Endocrinology, Department of Medicine, NYU Grossman School of Medicine, New York, NY, USA; 4 VA New York Harbor Healthcare System, Brooklyn, NY, USA

**Keywords:** lipids, triacylglycerol, ATGL, enzyme activity, NBD-TAG, lipoprotein lipase, lipolysis, orlistat, hormone-sensitive lipase, high-throughput screening, ApoCII, apolipoprotein CII, ATGL, adipose triglyceride lipase, DMSO, dimethylsulfoxide, FBS, fetal bovine serum, FFA, free fatty acid, HSL, hormone sensitive lipase, LpL, lipoprotein lipase, NBD, nitrobenzoxadiazole, PC, phosphatidylcholine, PI, phosphatidylinositol, PTL, pancreatic triglyceride lipase, TAG, triacylglycerol

## Abstract

Lipases constitute an important class of water-soluble enzymes that catalyze the hydrolysis of hydrophobic triacylglycerol (TAG). Their enzymatic activity is typically measured using multistep procedures involving isolation and quantification of the hydrolyzed products. We report here a new fluorescence method to measure lipase activity in real time that does not require the separation of substrates from products. We developed this method using adipose triglyceride lipase (ATGL) and lipoprotein lipase (LpL) as model lipases. We first incubated a source of ATGL or LpL with substrate vesicles containing nitrobenzoxadiazole (NBD)-labeled TAG, then measured increases in NBD fluorescence, and calculated enzyme activities. Incorporation of NBD-TAG into phosphatidylcholine (PC) vesicles resulted in some hydrolysis; however, incorporation of phosphatidylinositol into these NBD-TAG/PC vesicles and increasing the ratio of NBD-TAG to PC greatly enhanced substrate hydrolysis. This assay was also useful in measuring the activity of pancreatic lipase and hormone-sensitive lipase. Next, we tested several small-molecule lipase inhibitors and found that orlistat inhibits all lipases, indicating that it is a pan-lipase inhibitor. In short, we describe a simple, rapid, fluorescence-based triacylglycerol hydrolysis assay to assess four major TAG hydrolases: intracellular ATGL and hormone-sensitive lipase, LpL localized at the extracellular endothelium, and pancreatic lipase present in the intestinal lumen. The major advantages of this method are its speed, simplicity, and elimination of product isolation. This assay is potentially applicable to a wide range of lipases, is amenable to high-throughput screening to discover novel modulators of triacylglycerol hydrolases, and can be used for diagnostic purposes.

Lipases (triacylglycerol acyl hydrolases, EC 3.1.1.3) are enzymes produced by organisms including animals, plants, and microbes. They hydrolyze several insoluble triacylglycerols (TAGs) at the interface between hydrophobic substrates and water. Lipases play key roles as biotechnological catalysts in the manufacturing of many products including foods, detergents, cleaning agents, pharmaceuticals, paper, oil, gas, biopolymers, and textiles (1, 2). The growing industrial and therapeutic demand for lipases has resulted in the development of various spectrometric, radioactivity-based, chromatographic, and biosensor assays to determine their activities (3, 4, 5).

Lipases have several physiological functions in animals. In the intestinal lumen, they digest dietary TAGs, phospholipids, cholesteryl esters, and ester bonds in other lipids. In the circulation, they hydrolyze lipoprotein TAGs. In tissues, they hydrolyze stored TAGs. Hydrolysis of circulating and stored TAGs yields circulating free fatty acids (FFAs) that serve as energy sources for different organs such as the muscle, heart, and kidney (6).

Pancreatic triglyceride lipase (PTL) is produced in the pancreas in its active form and is secreted into the duodenum after hormonal stimulation by food ingestion. Its ability to break down bile-emulsified fats in the intestine is increased by colipase, another pancreatic protein. The FFAs produced are taken up by enterocytes (7, 8, 9). The intestine synthesizes chylomicrons to transport dietary fat, whereas the liver synthesizes very low density lipoproteins (VLDL) to transport endogenously synthesized fat. Both classes of lipoproteins enter the circulation, and endothelial cell surface anchored lipoprotein lipase (LpL) hydrolyzes chylomicron- and VLDL-associated TAG (10, 11, 12, 13, 14) in a process that is enhanced by apolipoprotein CII (apoCII), a cofactor of LpL, and results in the production of FFAs for tissue utilization. The pharmaceutical industry has developed methods to either block the activity of PTL in the gastrointestinal tract, or the activity of several circulatory inhibitors of LpL as a means to enhance LpL activity, both in order to reduce circulatory TAG levels (15).

Adipocytes take up and re-esterify FFAs and store them as TAGs. When caloric intake is reduced, intracellular lipases break the ester bonds between FAs and glycerol to mobilize FFAs as energy source. This hydrolysis occurs in three steps involving three different enzymes (6, 16, 17). First, adipose triglyceride lipase (ATGL) initiates the hydrolysis of TAG, producing diacylglycerol and FFA. Next, hormone-sensitive lipase (HSL) hydrolyzes diacylglycerol into monoacylglycerol and FFA. HSL can act on both TAG and diacylglycerol, but it is 10 times more specific to diacylglycerol compared with TAG (18). Subsequently, monoacylglycerol lipase hydrolyzes monoacylglycerol into FFA and glycerol. Lipolysis is highly regulated, and defects in the process lead to either obesity or lipodystrophy. Studying these enzymes requires measurement of their activities, as they are regulated by posttranslational modifications involving the phosphorylation of HSL and the association of ATGL with other proteins (19, 20, 21, 22, 23).

Several methods exist to measure lipase activities (24, 25, 26), but they are time-consuming and require a step for separating products from substrates. For instance, continuous titration with a pH-stat coupled to an automatic burette and recorder is considered to be the reference method to study PTL kinetics; however, it involves specific instrumentation and expertise (27, 28). Colorimetric and fluorometric assays using either diacylglycerol or TAG substrates have been developed but have limited use in the research setting due to inflexibility of the assay conditions (27). For LpL, fluorescence-based assays have been described, of which the most recent uses EnzCheck substrate solubilized in Zwittergent (3). This substrate contains BODIPY-C12 FA at the sn-1 position and a fluorescence quencher at the sn-2 position. Hydrolysis of the substrate by LpL releases BODIPY, and the resulting increase in fluorescence is measured to represent LpL activity. However, this substrate becomes unquenched when mixed with many detergents. Basu *et al.* (3) reported that the EnzCheck remains quenched in Zwittergent and that LpL hydrolyzes EnzCheck in a time-, substrate-, and protein-dependent manner.

ATGL activity, in contrast, is generally measured using radiolabeled substrates (25, 29): [^3^H]triolein is emulsified in phospholipids and incubated with tissue or cell lysates, and then FFA are extracted and counted. However, the use of radioactive substrate limits the suitability of this assay for high-throughput screening. Attempts have been made to measure ATGL activity using fluorogenic substrates (30) such as dibutyrilfluoresceine (31), parinaric acid (32, 33), umbelliferone (34, 35), resorufin (24), pyrenic compounds, and BODIPY-C12 (4, 36, 37, 38). These assays have some disadvantages, namely the compounds are spontaneously hydrolyzed at pH 8.8, are susceptible to oxidation by atmospheric oxygen (39), and are poorly hydrolyzed by lipases (38, 40). Nitrobenzoxadiazole (NBD) is a better fluorophore because it is small, does not interact with colored compounds, and is insensitive to oxidation (41, 42). More importantly, TAG and phospholipid synthesizing enzymes recognize NBD-fatty acids (43, 44, 45). Muller *et al.* (46) used NBD-fatty acid to assess lipolysis in adipocytes. In their assay, adipocytes were incubated with NBD-fatty acid to label intracellular lipid droplets, washed, and stimulated to induce lipolysis, and NBD-fatty acid released into the medium was quantified to assess lipolysis. This procedure has the drawback of requiring cell labeling followed by stimulation with various receptor agonists. In addition, it cannot be used to assess lipolysis in nonadipose cells, which do not store significant amounts of TAG and do not respond to adrenergic receptor agonists.

We have used NBD-labeled lipids previously to develop TAG, cholesteryl ester, and phospholipid transfer assays for microsomal triglyceride transfer protein (47, 48). To date, NBD-labeled TAG (NBD-TAG) has not been used to assess the enzymatic activities of ATGL, HSL LPL, or PTL. Therefore, we examined the utility of NBD-TAG as a possible substrate to measure lipases and developed a simple, rapid fluorescence-based assay to quantify the lipolytic activities of these enzymes. This assay can be used in high-throughput screening of lipases to discover novel inhibitors and activators.

## Materials and methods

### Materials

Egg phosphatidylcholine (PC, #131601) and phosphatidylinositol (PI, #P0639) were purchased from Avanti Lipids and Millipore Sigma, respectively. Nitrobenzoxadiazole-labeled TAG [(NBD-TAG; 1,3-di(*cis*-9-octadecenoyl)-2-((6-(7-nitrobenz-2-oxa-1,3-diazol-4-yl)amino)hexanoyl)glycerol)] (#6285) and NBD-C6 (6-(*N*-(7-nitrobenze-2-oxa-1,3-diazol-4-yl)amino)hexanoic acid) (#6867) were obtained from Setareh Biotech. DMEM (Dulbecco's Modified Eagle's Medium with 4.5 g/L glucose, L-glutamine, and sodium pyruvate, #2021-05) and phosphate-buffered saline (without calcium and magnesium, #21-040-CM) were from Corning. Fetal bovine serum (FBS) (#10437-028) and penicillin-streptomycin (5,000 U/ml, #15070063) were procured from Gibco. EndoFectin™-Max (#EF013) was acquired from GeneCopoeia. The ATGL inhibitor atglistatin (#530151) was bought from Millipore Sigma and dissolved in dimethylsulfoxide (DMSO) to prepare a 10 mM stock. Orlistat (#10005426) was purchased from Cayman Chemicals, and a 20 mM stock was prepared in DMSO. The HSL inhibitor 2-(2-ethoxycarbonylphenyl)-5,5-dimethyl-1,3,2-dioxaborinane (#SC206328) was purchased from Santa Cruz Biotechnology at 1.09 g/ml. Colipase from porcine pancreas (#C3028), BICINE (catalog # B3876), deoxycholic acid (#D2510), and calcium chloride (#C3306) were all obtained from Sigma. Anti-His antibody was procured from Abcam (#ab5000) and used at a 1:1,000 dilution in 2% bovine serum albumin (BSA). Secondary anti-mouse antibody was purchased from Invitrogen (#62-6520) and used at a 1:5,000 dilution in 2% BSA. Plasmids expressing human (hATGL), mouse (mATGL) ATGL, mouse HSL (mHSL), and a control LacZ-expressing pcDNA3 plasmid, were gifts from Dr Rudolf Zechner of the University of Graz (Austria).

### Expression of enzymes in Cos-7 cells

Cos-7 cells (0.8 million) were seeded in 60 mm dishes and cultured in DMEM containing 10% FBS and 1% antibiotic solution. After 24 h, cells received Opti-MEM medium and were transfected with 7.5 μg of pcDNA3 (control plasmid) or plasmid expressing hATGL, mATGL, or mHSL (29) using EndoFectin (2.5 μl per μg of plasmid). After 14–16 h of transfection, the medium was changed to DMEM containing 10% FBS without antibiotics. After 48 h, cells were washed with ice-cold PBS and harvested in 1 ml of buffer K (1 mM Tris-HCl, pH 7.6, 1 mM EGTA, and 1 mM MgCl_2_) containing 10 μl/ml of protease inhibitor cocktail (#P2714, Sigma) in preparation for homogenization.

### Preparation of cell lysates and detection of expressed proteins

Cells were homogenized in ice-cold buffer K by passage through a 27 1/2 G needle attached to a 1 ml syringe 15–20 times. The homogenized cells were centrifuged at 13,523 *g* (12,000 rpm, Eppendorf centrifuge 5424 R) for 10 min at 4°C. The supernatant was collected, and protein content was estimated using bicinchoninic acid method (# 23228, Thermo Fisher Scientific). Equal amounts of proteins were resolved on an 8% SDS–polyacrylamide gel. Targeted protein expression was analyzed by immunoblotting using specific antibodies. Anti-His antibody was procured from Abcam (#ab5000). Primary antibody was used at a 1:1,000 dilution in 2% BSA. Horseradish peroxidase–conjugated secondary anti-mouse antibody was purchased from Invitrogen (#62-6520) and used at 1:5,000 dilution in 2% BSA. Horseradish peroxidase–conjugated secondary antibody was detected using chemiluminescence detector (#37069, Thermo Scientific). The blot was imaged using a Bio-Rad ChemiDoc™ Touch Imaging System.

### Electron microscopy

Vesicles were negatively stained with uranyl acetate (49). Briefly, formvar/carbon-coated grids were floated carbon side down onto a drop of the liposome liquid for 30 s, washed twice in double distilled water, and negatively stained on a drop of aqueous 0.5% uranyl acetate for 60 s. The grid was then blotted, dried overnight in the dark, and analyzed the next day with a Zeiss EM900 transmission electron microscope retrofitted with a SIA L3C digital camera (SIA, Duluth, GA). Vesicles were measured utilizing Maxim DL software (Diffraction Limited, Ontario, Canada).

### Purification of bovine milk LpL

LpL was partially purified from fresh unpasteurized cow's milk using heparin affinity chromatography (50). The milk was first spun to remove the cream, and NaCl (∼29 g/L) was added to the skim milk to obtain a concentration of 0.5 M NaCl. Heparin-agarose (50 ml) was added, and the solution rocked at 4°C for 3 h. The gel was centrifuged at 5,000 rpm for 10 min, washed with ice-cold 0.5 M and then 1.0 M NaCl in PBS, and incubated with 25 ml of 2 M NaCl. The released LpL in 2 M NaCl was aliquoted, stored at –70°C, and defrosted before use.

### Preparation of NBD-TAG-containing substrates

NBD-TAG stock was prepared by dissolving 10 mg of NBD-TAG in 1 ml chloroform and stored at –80°C. Purchased egg PC stock was 25 mg/ml. To prepare NBD-TAG vesicles, 32 μl (352 nmol) of NBD-TAG stock was mixed with 69.2 μl (2,208 nmol) of PC stock in 5 ml glass bottle. The mixture was dried using nitrogen in an evaporator (Organomation N-EVAP™ 112). The dried lipid coating was suspended in 4 ml of buffer II (15 mM Tris, pH 7.4%, 0.02% sodium azide, 1 mM EDTA, 40 mM NaCl) and sonicated (Sonic Dismembrator 550 from Fisher Scientific) on ice for 30 min until the mixture was clear (51). The clear solution was subjected to ultracentrifugation at 233,800 *g* (50,000 rpm, SW50.1 Ti rotor) for 1 h at 10°C. The top 4 ml of NBD-TAG vesicles were collected, 200 mg BSA and 650 mg NaCl were added, and the vesicles were stored at 4°C until use. At 4°C, the prepared vesicles were stable for at least 4 months. Total and background fluorescence was measured in 10 μl of vesicles after adding 90 μl isopropanol (total) or buffer K (background or blank). The background fluorescence was ∼10% of the total.

PI stock was prepared by dissolving 20 mg PI in 800 μl of chloroform. To prepare PC:PI vesicles containing NBD-TAG, 32 μl (352 nmol) of NBD-TAG stock was mixed with 69.2 μl (2,208 nmol) of PC stock and 23 μl (776 nmol) of PI to obtain a 3:1 M ratio between PC and PI. The molar ratio between TAG:PC:PI was 1:6:2. The mixture was evaporated under nitrogen, suspended in 4 ml of buffer II, sonicated, and centrifuged as described above for PC vesicles. The top 4 ml of NBD-TAG in PC/PI vesicles was collected, 200 mg of BSA and 650 mg of NaCl were added, and the vesicles were stored at 4°C. The background fluorescence was ∼10%. These vesicles were stable for at least 4 months at 4°C.

To prepare emulsions containing TAG and PC at a ratio of 7:1, NBD-TAG (114 μl, 1262.8 nmol), PC (5.5 μl, 176 nmol), and PI (2 μl, 59 nmol) were evaporated, suspended in 4 ml of buffer II, sonicated, and centrifuged as described above. The top 4 ml of NBD-TAG in PC/PI vesicles was collected, 200 mg of BSA and 650 mg of NaCl were added, and the vesicles were stored at 4°C. These vesicles were stable for at least 4 months under these conditions. The background fluorescence in these vesicles was ∼2%.

### Preparation of a NBD-C6 standard curve

NBD-C6 (2.94 mg) was dissolved in 1 ml of DMSO to obtain a 10 mM stock solution. The stock was diluted in buffer K to get working concentrations of 1,250, 625, 312.5, 125, 31.25, and 12.5 pmol in 100 μl buffer K. The NBD fluorescence readings were measured at excitation 460 nm and emission 530 nm wavelength settings using a Victor^3^ 1420 multi-label counter from PerkinElmer and plotted against NBD-C6 concentrations. Background values in solutions containing no NBD were subtracted.

### Lipolysis assay

With NBD-TAG in PC vesicles as substrate, the assays were performed in triplicate in 2 ml opaque centrifugation tubes (Fisher Scientific, #02-681-291) resistant to organic solvents. For enzyme activity measurements, NBD-TAG in PC vesicles (10 μl, 880 pmol of NBD-TAG) were incubated with cell homogenates (100 μg protein in a total volume of 200 μl) in a 37°C incubator (Precision Thelco laboratory). After 1 h, the samples were extracted through the addition of 1 ml of solvent mixture A (10:9:7 (v/v/v) methanol/chloroform/*n*-heptane) and 400 μl of buffer B (0.1 M potassium carbonate, pH 10.5) to the centrifugation tube as described by Schweiger *et al.* (25). The samples were mixed vigorously using a vortex and subjected to low-speed centrifugation at 1,000 *g* (2,278 rpm, Eppendorf 5415D centrifuge) for 10 min (52). The upper, aqueous phase (200 μl) was transferred to an opaque round-bottom 96-well assay plate (#3792, Costar), and fluorescence was measured with excitation 460 nm and emission 530 nm wavelength settings using a Victor^3^ 1420 multi-label counter from PerkinElmer. These fluorescence readings were used to calculate the FFAs released using the NBD-C6 standard curve prepared in parallel as described above.

With NBD-TAG in PC/PI vesicles, lipase assays were performed in triplicate in opaque round-bottom 96-well assay plate (#3792, Costar). The plate wells contained a series of NBD-C6 dilutions representing a standard curve ranging from 0 to 1,250 pmol as described above. For background fluorescence measurements, NBD-TAG in PC/PI vesicles (10 μl, 880 pmol of NBD-TAG) was incubated with 100 μl of buffer K without any enzyme source, and these fluorescence values were subtracted from all values. For lipase assay, substrate vesicles (10 μl, 880 pmol of NBD-TAG) were incubated with 100 μl of buffer K containing different amounts of enzyme source. Plates were incubated at 37°C or at room temperature for different time periods, and fluorescence was measured using excitation 460 nm and emission 530 nm wavelengths. Background fluorescence was subtracted from lipase assay wells. The background represents unquenched fluorescence from NBD-TAG in the vesicles and is usually less than 10% of the total fluorescence present in these vesicles, as determined after dissolution of vesicles with isopropanol (47, 48). The fluorescence values were then used to quantify amounts of NBD-C6 released using the standard curve. Data are presented as NBD-C6 released, or as specific activity, where NBD-C6 released was normalized to 1 mg of protein and 1 h of incubation.

Using 7:1 NBD-TAG to PC/PI emulsions, lipase assays were performed in triplicates, as described above for NBD-TAG in PC/PI vesicles with a ratio of 1:6. Briefly, either 3 μl (880 pmol of NBD-TAG) for comparison with earlier vesicles or 10 μl of substrate (3,155 pmol of NDB-TAG) was incubated with different enzymes at room temperature, and fluorescence was measured at different time points. Background fluorescence was subtracted, and the NBD-C6 released was quantified using a standard curve.

### Measurement of PTL activity in pancreas

We collected the pancreas from fed C57BL/6 mice and snap froze in liquid nitrogen. Frozen tissue was crushed using a cryo-cup grinder with pestle (Fisher Scientific, #50550167) in liquid nitrogen to prevent the tissue from thawing while grinding. The powdered tissue was homogenized in modified buffer K (50 mM Tris-HCl, pH 8, 1 mM EGTA, and 50 mM MgCl_2_) containing 1 tablet of ml protease inhibitor (Sigma, #11836170001) per 7 ml of buffer and centrifuged at 13,523 *g* (12,000 rpm, Eppendorf centrifuge 5424 R) for 10 min at 4°C. We then carefully collected the clear supernatant. Protein levels were measured, and 40 μg of tissue homogenates, in the presence and absence of different amounts of colipase, was used for PTL activity measurements as described above. Modified buffer K with different amounts of colipase in solution was prepared to serve as blanks. A colipase solution containing 0.98 mg colipase, 1.6 mmol deoxycholic acid, and 10 mmol CaCl_2_ per liter of Bicine buffer (50 mmol/L, pH 8) was prepared following instructions for a colorimetric lipase reagent formulation by Roche Custom Biotech. This stock was used to obtain desired amounts of colipase.

### Measurement of lipase activities in the presence of different inhibitors

Atglistatin is a competitive mATGL inhibitor with IC_50_ = 0.7 μM (53). It inhibits mATGL without displacing it from the lipid droplet surface (53). Atglistatin selectively inhibits FFA release from murine 3T3L1 cells, but not from human cells (54). Orlistat inhibits gastric, pancreatic, and carboxyl ester lipases and is used to treat and manage obesity. Orlistat acts by binding covalently to the serine residue of the active site of gastric and pancreatic lipases (55, 56). We used 100 μM atglistatin, 20 μM orlistat, and 20 μM of the HSL inhibitor SC206328. Cos-7 cell homogenates expressing LacZ, mATGL, hATGL, or mHSL were incubated with NBD-TAG vesicles in the presence and absence of the three inhibitors. To measure inhibition of LpL activity, purified bovine milk LpL and 5% mouse plasma were incubated with NBD-TAG vesicles in the presence and absence of inhibitors. To measure the inhibition of PTL activity, pancreatic tissue homogenates with 80 ng of colipase in solution were incubated with NBD-TAG vesicles in the presence and absence of the three inhibitors. For background fluorescence, buffer K (or modified buffer K for the pancreas) was incubated with NBD-TAG vesicles in the presence and absence of the inhibitors. NBD fluorescence was measured at different time intervals. Background fluorescence was subtracted from the sample readings, and FFA released was quantified using the NBD-C6 standard curve.

### Measurement of ATGL and HSL activity in eWAT of mice during fed and fasting conditions

C57BL/6 mice were purchased from Jackson laboratory and maintained on chow diet (PicoLab Rodent diet 20 #5053 from Lab Diet) at NYU Long Island School of Medicine animal facility. Institutional animal care and use committee approved all experimental protocols. Male 20-week-old mice were euthanized under deep anesthesia at 10 AM by cervical dislocation, and transcardial perfusion was performed with normal saline before dissecting different organs. The organs were snap frozen in liquid nitrogen and kept at –80°C until use. In fasting experiments, the mice were fasted overnight (16 h), euthanized at 10 AM to collect tissues. We collected epididymal white adipose tissue (eWAT) from fed and 16 h fasted C57BL/6 mice. Tissues were homogenized in buffer K and centrifuged at 13,523 *g* (12,000 rpm, Eppendorf centrifuge 5424 R) for 10 min at 4°C. We carefully collected the middle clear portion without disturbing the floating fat layer. This portion was centrifuged again to minimize contamination from endogenous tissue lipids. Protein levels were measured, and 100 μg of tissue homogenates was used for ATGL and HSL activity measurements as described above.

### Statistics

We performed statistical analysis using GraphPad Prism versions 8 and 9. All data are represented as mean ± SD. The symbols ∗, ∗∗, ∗∗∗ represent significance at *P* < 0.05, *P* < 0.01, and *P* < 0.001, respectively. The percent coefficient of variation (CV) was calculated based on equation % CV = (SD/average) × 100.

## Results

### Preparation of an NBD-C6 reference standard curve

**Fig. 1:**
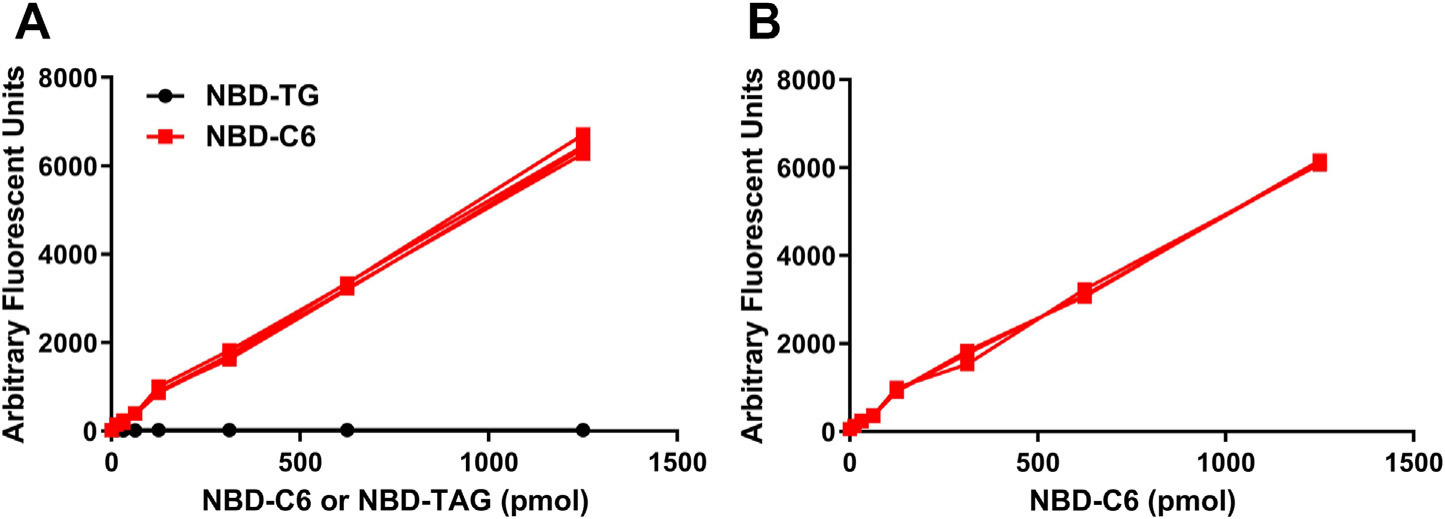
Separation of NBD-C6 from NBD-TAG. A: The different amounts of NBD-C6 (n = 4) and NBD-TAG (n = 4) were incubated separately in buffer K at 37°C for 1 h and subjected to lipid extraction as described in Materials and methods. NBD fluorescence in top, aqueous phase was measured. B: Different amounts of NBD-C6 (n = 3) were mixed with a fixed amount of NBD-TAG (1,250 pmol) and incubated in buffer K at 37°C for 1 h. NBD fluorescence in aqueous phase was measured after lipid extraction. Data representative of four independent experiments.

Lipases hydrolyze TAG to produce FFAs and diacylglycerol (29, 57, 58, 59). To develop a fluorescence-based assay for lipases, we used NBD-TAG, which we previously used to develop TAG transfer assays for MTP (47, 48). The NBD-TAG contains NBD-C6 at its sn-2 position and is expected to be released after hydrolysis. To quantify released NBD-C6, we developed a reference standard curve using commercially available NBD-C6. Another consideration during the development of this assay was that the substrate NBD-TAG should not interfere with measurements of the product NBD-C6. For this purpose, we used well-described methods of separating TAGs from FFAs based on differential solubility in polar and nonpolar solvents (52) that were previously used in radiolabel assays to measure ATGL activity (25). To ascertain the adequate separation of NBD-C6 from NBD-TAG and establish a reference standard curve, we first incubated different amounts of NBD-C6 and NBD-TAG separately at 37°C for 1 h in 200 μl of buffer K, then subjected them to the extraction procedure described in Methods. We measured sample fluorescence in a different plate after transferring 200 μl per well of supernatant. Fluorescence increases were linear with the concentrations of NBD-C6 used (Fig. 1A) and did not show saturation, suggesting that the amounts of solvents used for extraction are in excess. The recovery of NBD-C6 was 98%. When samples containing increasing concentrations of NBD-TAG were extracted, there was no detectable fluorescence, indicating that NBD-TAG was not extracted in the aqueous phase (Fig. 1A). We next mixed NBD-TAG with NBD-C6 and subjected the mixture to the same extraction procedure. Again, recovery of NBD-C6 was close to 98% (Fig. 1B) suggesting that NBD-C6 was quantitatively extracted in the presence of TAG. These results confirmed that the extraction method effectively separates NBD-TAG from NBD-C6 and that these conditions are suitable for use in developing lipase assays. Further concentration-dependent increases in fluorescence of NBD-C6 ensure quantitative assessment of the hydrolyzed products over a wide range of concentrations.

### Effect of protein concentration on the hydrolysis of NBD-TAG in PC vesicles

**Fig. 2:**
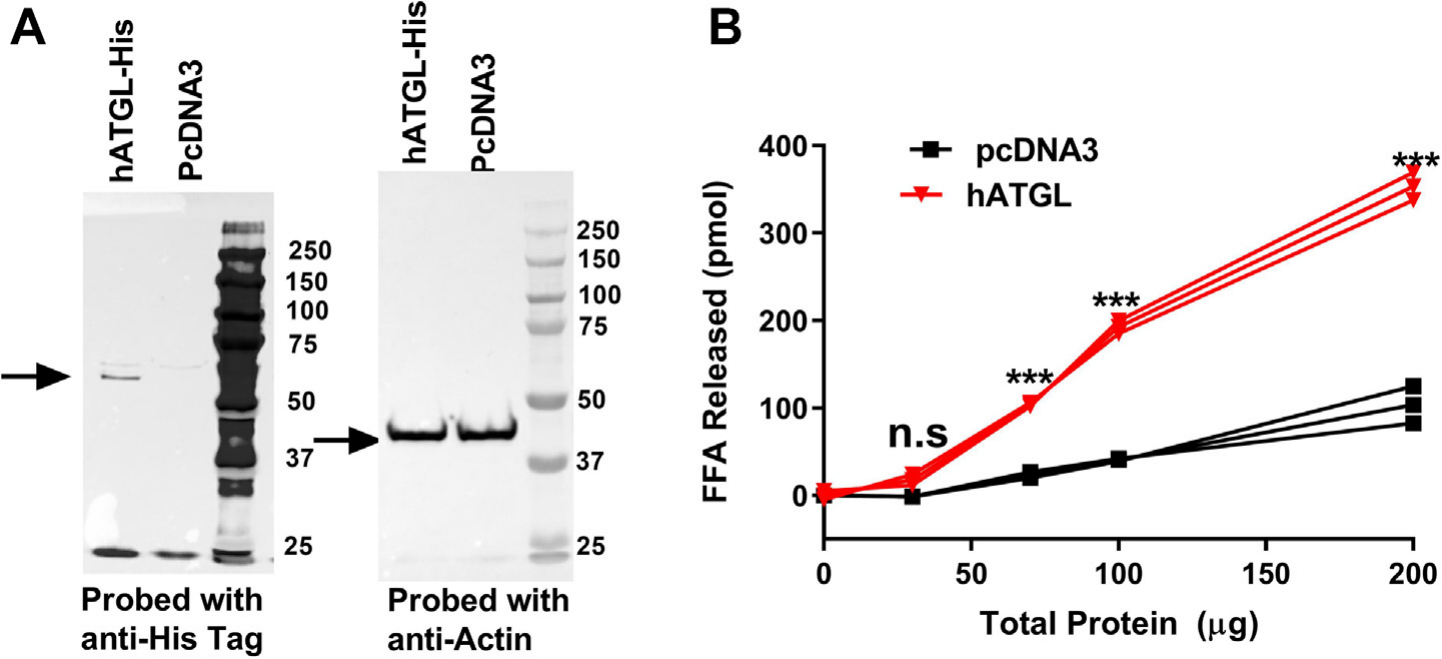
hATGL protein-dependent hydrolysis of NBD-TAG. A: Cos-7 cells were transfected in triplicate with the plasmid pcDNA3 or pcDNA3 expressing His-tagged human ATGL (hATGL). Cells were collected and homogenized in buffer K as described in Methods. Cell lysates (20 μg protein) were separated on gels and probed with anti-His antibodies (left) and actin (right). B: Different amounts of cell lysate proteins were incubated with NBD-TAG (10 μl, 880 pmol) in PC vesicles for 1 h. Fluorescence in the aqueous phase was measured after lipid extraction. The amounts of NBD-C6 were quantified using a standard curve as described in Fig. 1. Amounts of NBD-C6 released were plotted against the amounts of protein used. N = 3; significance was calculated using two-way ANOVA followed by Bonferroni's post-test analysis. ∗∗∗*P* < 0.001; ns, not significant.

To develop an assay for ATGL, we transfected monkey kidney Cos-7 cells with a plasmid encoding His-tagged hATGL (29). As a control, cells were transfected with empty pcDNA3 vector. We then measured total cell protein and hATGL expression in cell lysates (Fig. 2A). To measure ATGL activity, we incubated different amounts of cell lysates with fixed amounts of NBD-TAG-containing PC vesicles (prepared using a 1:6 M ratio of NBD-TAG to PC; Fig. 2B) and then extracted FFAs after 1 h. We detected protein concentration-dependent increases in NBD fluorescence, but the increase was significantly (6-fold) higher in hATGL-expressing cell lysates compared with the control pcDNA3-transfected cells (Fig. 2B). These studies demonstrated that higher concentrations of proteins from hATGL-expressing cells hydrolyze more NBD-TAG substrate.

### Effect of substrate concentration and time course of NBD-TAG hydrolysis by hATGL

**Fig. 3:**
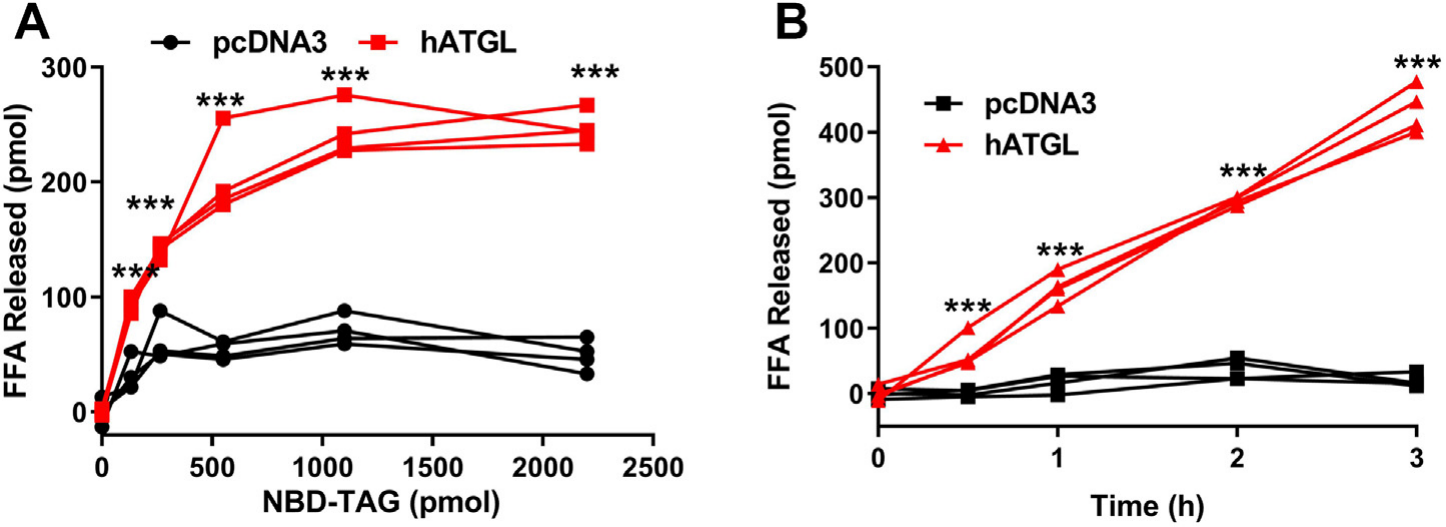
Effect of different substrate concentrations and time course of NBD-TAG hydrolysis by hATGL. A: hATGL-expressing or pcDNA3-control plasmid transfected Cos-7 cell lysates (100 μg protein) were incubated (n = 4) with different amounts of NBD-TAG for 1 h. After lipid extraction, fluorescence readings were measured and NBD-C6 levels were quantified using a standard curve. B: Cell lysates (100 μg protein) from hATGL-expressing or pcDNA3-control plasmid transfected Cos-7 cells were incubated (n = 4) with 10 μl (880 pmol) of NBD-TAG for different times. After lipid extraction, fluorescence readings were measured. Individual values were plotted. Significance was calculated using two-way ANOVA followed by Bonferroni's post-test analysis. ∗∗∗*P* < 0.001.

To understand the effects of different substrate concentrations on hATGL activity, we incubated fixed amounts of cell lysates from Cos-7 cells transfected with the hATGL expression plasmid or pcDNA3 with different amounts of NBD-TAG in PC vesicles (Fig. 3A). Lysates from hATGL-expressing cells showed increased NBD-C6 release with increasing substrate concentrations, whereas lysates from control pcDNA3-transfected Cos-7 cells showed low hydrolysis that saturated at a lower concentration (Fig. 3A). At higher NBD-TAG concentrations, there was no further increase in NBD-C6. At saturation, the lysates from hATGL-expressing cells showed 4–5 times more NBD-C6 release than those from pcDNA3-transfected cells. Thus, the hydrolysis of NBD-TAG shows saturation kinetics with increasing concentrations of substrate.

To determine the optimum time required to measure ATGL activity, we next incubated the cell lysates with NBD-TAG vesicles for different periods (Fig. 3B). TAG hydrolysis increased significantly over time with cell lysates obtained from hATGL-expressing cells, but not with those from control pcDNA3-transfected cells. The amounts of FFAs released by hATGL-expressing cell lysates were significantly higher (∼5 times) than those for pcDNA3-transfected cell lysates (Fig. 3B). These studies demonstrated that substrate hydrolysis increases with time and that 1 h incubation is sufficient to measure ATGL activity.

### Inclusion of phosphatidylinositol in substrate vesicles enhances NBD-TAG hydrolysis and abolishes the need to extract free fatty acids for quantifications

**Fig. 4:**
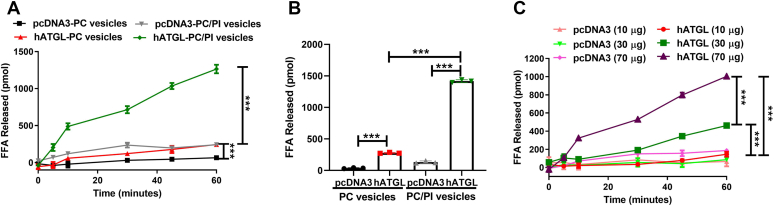
Effect of time course and protein concentrations on the hATGL activity using NBD-TAG in PC/PI vesicles. A and B: Comparison of PC and PC/PI vesicles as substrates. pcDNA3 or hATGL transfected Cos-7 cell lysates (100 μg protein) were incubated (n = 3) with either PC vesicles or PC/PI vesicles containing NBD-TAG (10 μl, 880 pmol) for 1 h. A standard curve of NBD-C6 was prepared in parallel to quantify released FFAs. A: NBD fluorescence was measured at 0, 5, 10, 30, 45, and 60 min and FFA release was calculated using NBD-C6 standard curve. B: After 1 h incubation, FFAs were extracted as described earlier and NBD fluorescence readings were measured to calculate FFA concentrations. C: Effect of time and protein concentrations on substrate hydrolysis. Different amounts of lysate proteins from Cos-7 cells transfected with either pcDNA3 or hATGL expression plasmid were incubated with NBD-TAG (10 μl, 880 pmol) containing PC/PI vesicles. Fluorescence readings were taken at different intervals to measure time course of hydrolysis. Error bars represent SD. For A and C, significance was calculated using two-way ANOVA followed by Bonferroni's post-test analysis. For B, one-way ANOVA was performed followed by Bonferroni's post-test analysis. ∗∗∗*P* < 0.001. The experimental results are representative of two or three independent experiments.

Two drawbacks of the assay described above are that it requires extraction of FFAs and is relatively insensitive. The extraction was necessary because of the low amounts of FFAs released and to avoid background contributions from NBD-TAG. The FFA extraction step hinders the development of a high-throughput assay to identify novel ATGL inhibitors. We hypothesized that optimizing hATGL activity assay conditions to increase product to substrate ratio might avoid the need to extract FFAs. Inclusion of phosphatidylinositol (PI) during the assay can enhance ATGL activity (25). Therefore, we prepared new PC and PI (PC/PI) vesicles containing NBD-TAG and compared them with NBD-TAG vesicles containing PC only as substrates for hATGL activity (Fig. 4). The lysates from hATGL-expressing Cos-7 cells showed significantly more FFA release with time than lysates from pcDNA3-transfected cells when either PC or PC/PI vesicles were used (Fig. 4A). More importantly, the lysates from hATGL-expressing cells showed seven times more FFA release from PC/PI vesicles compared with PC vesicles (Fig. 4A), indicating that inclusion of PI significantly enhances hydrolysis of NBD-TAG. To make sure that we were measuring released FFAs and not NBD-TAG, at the end of the assay, we extracted FFAs and measured fluorescence (Fig. 4B). This confirmed that the amounts of FFAs released were significantly greater when hATGL was incubated with NBD-TAG in PC/PI vesicles. These studies showed that NBD-TAG in PC/PI vesicles is a better substrate for hATGL than NBD-TAG in PC-only vesicles. More importantly, the FFA concentrations calculated without extraction of FFAs were similar to those obtained after FFA extraction, indicating that FFA extraction is not necessary to measure hATGL activity using PC/PI vesicles. We therefore used these NBD-TAG-containing PC/PI vesicles in subsequent studies. Moreover, we used increases in fluorescence units to calculate amounts of FFAs released using a standard curve of NBD-C6 without lipid extraction, unless otherwise stated.

We then studied the effect of protein concentration on hATGL activity over time using NBD-TAG in PC/PI vesicles (Fig. 4C). Only very small amounts of FFAs were released when we used low (10 μg) concentrations of proteins from either hATGL-expressing or control pcDNA3-transfected cells, consistent with the results in Fig. 2. Higher concentrations of proteins from hATGL-expressing cells released significantly higher amounts of FFAs (Fig. 4C), although using higher concentrations of proteins from pcDNA3-transfected cells had no effect (Fig. 4C), indicating that the increase in hATGL led to the enhanced substrate hydrolysis. These results confirmed that PC/PI vesicles are better than PC-only vesicles for measuring ATGL activity and are consistent with earlier studies (21).

### Effect of substrate concentration and temperature on hATGL activity detected using NBD-TAG in PC/PI vesicles

**Fig. 5:**
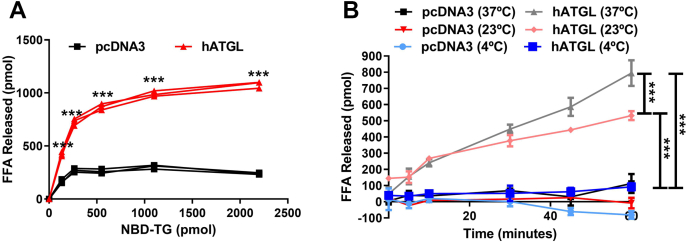
Effect of substrate concentrations and temperatures on hATGL activity. A: hATGL-expressing or pcDNA3-control plasmid transfected Cos-7 cell lysates (100 μg protein) were incubated with different amounts of NBD-TAG in PC/PI substrate vesicles for 1 h. A: Fluorescence was measured after 1 h incubation. FFAs released were quantified using NBD-C6 standard curve. B: hATGL-expressing or pcDNA3-control plasmid transfected Cos-7 cell lysates (70 μg protein) were incubated with PC/PI NBD-TAG (10 μl, 880 pmol) substrate vesicles for 1 h at different temperatures. NBD fluorescence readings were taken at different time intervals during incubation, and amounts of NBD-C6 released were quantified. Error bars represent SD, n = 3. Significance was calculated using two-way ANOVA followed by Bonferroni's post-test analysis; ∗∗∗*P* < 0.001. The results are representative of three to five independent experiments.

With increasing substrate concentrations of NBD-TAG in PC/PI vesicles, lysates from hATGL-expressing cells showed significant increase in fluorescence that reached saturation (Fig. 5A). In contrast, we detected only a small increase in FFAs released when we incubated pcDNA3-transfected cell lysates with increasing concentrations of substrates. At saturation, NBD-C6 production by ATGL-expressing Cos-7 cell lysates was six times higher than for control pcDNA3-transfected Cos-7 cell lysates. Thus, hydrolysis of the NBD-TAG by ATGL-expressing cells was more robust in PC/PI vesicles than pcDNA3-transfected cells and exhibited saturation kinetics.

Next, we studied the effect of temperature on hATGL-mediated TAG hydrolysis. Fluorescence readings did not increase with time when cell lysates from hATGL-transfected cells were incubated with NBD-TAG in PC/PI vesicles at 4°C, or for cell lysates from pcDNA3-transfected cells at any temperature (Fig. 5B). In contrast, incubating cell lysates from hATGL-expressing cells with the vesicles at either 23°C or 37°C resulted in time-dependent increases in fluorescence that were more pronounced at 37°C (Fig. 5B). These results indicate that hATGL is active at both higher temperatures and that its activity is temperature dependent.

### Lipolytic activity of human and mouse ATGL and effect of inhibitors

**Fig. 6:**
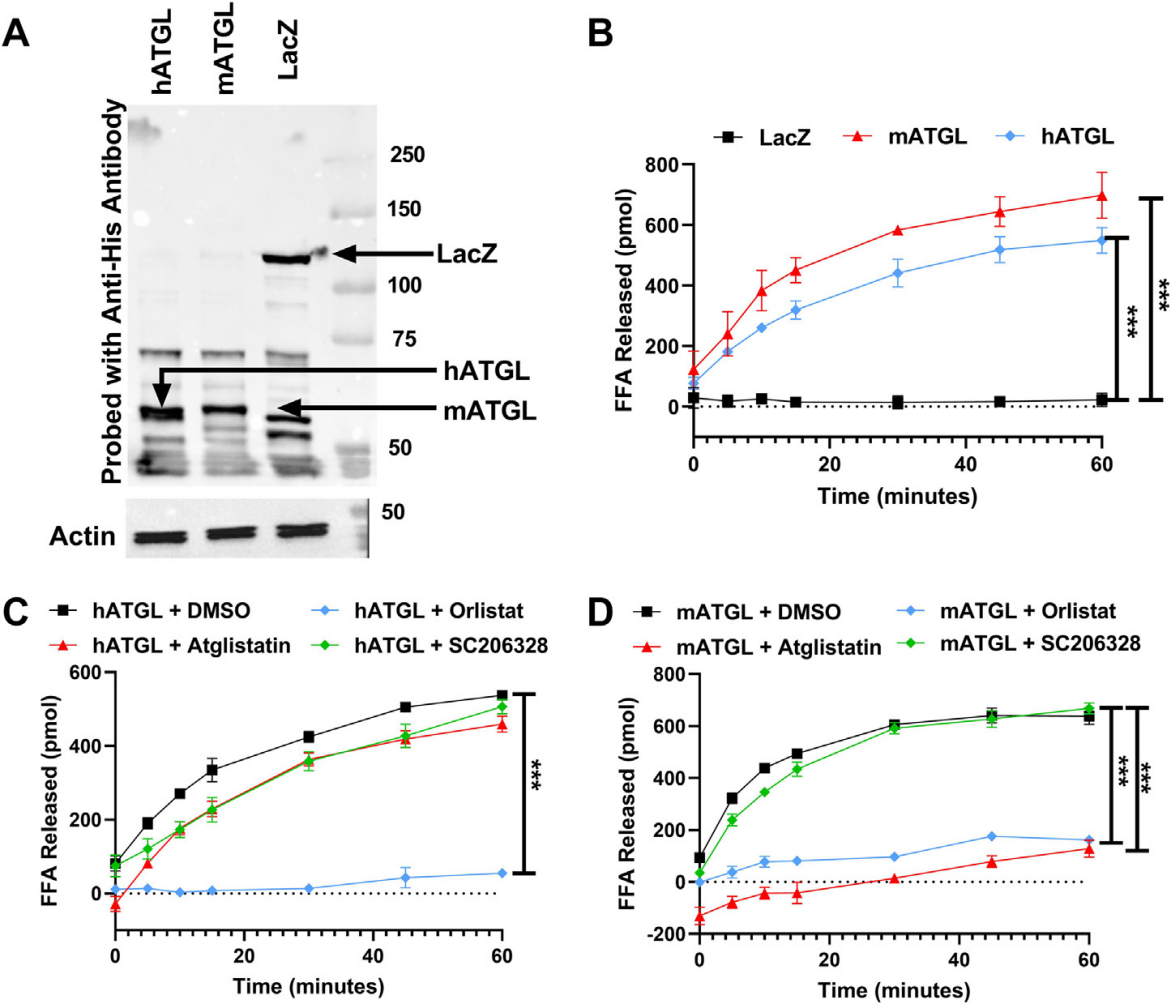
Effect of different inhibitors on hATGL and mATGL. A: Cos-7 cells were transfected with plasmids expressing His-tagged LacZ, mATGL, or hATGL. Cell lysates (20 μg) were separated on a gel and probed with anti-His antibodies (top). In another gel, actin was probed to show equal loading of cell proteins in different wells (bottom). B: LacZ-, mATGL-, or hATGL-expressing Cos-7 cell lysates (50 μg) were incubated with NBD-TAG (10 μl, 880 pmol) substrate for 1 h. Fluorescence was measured at different time intervals during incubations to calculate FFA released. Significance was calculated using one-way ANOVA followed by multiple comparisons test. Error bars represent SD. C: hATGL and (D) mATGL transfected Cos-7 cell lysates (50 μg) were incubated with NBD-TAG (10 μl, 880 pmol) substrate in presence of either DMSO, atglistatin (100 μM), orlistat (20 μM), or SC206328 (20 μM). Fluorescence was measured at different time points during the 1 h incubation to calculate FFAs released. The graphs are representative of three to seven independent experiments, each with three biological replicates. Error bars represent SD; ∗∗∗*P* < 0.001 as calculated by two-way ANOVA followed by multiple comparisons test.

To check how different inhibitors affect the activity of hATGL and mATGL, we first expressed His-tagged LacZ (control), mATGL, and hATGL in Cos-7 cells (Fig. 6A). The cells expressing mATGL or hATGL showed robust increases in FFAs released compared with cells expressing LacZ (Fig. 6B). To understand the effects of three different inhibitors, atglistatin, hormone-sensitive lipase inhibitor (HSLi) (SC206328), and orlistat, we incubated cell lysates expressing mATGL and hATGL with each inhibitor. The activity of hATGL was inhibited by orlistat but not by atglistatin or HSLi (Fig. 6C). Both atglistatin and orlistat significantly diminished FFAs release by mATGL, but HSLi had no effect (Fig. 6D). These results are consistent with other reports that atglistatin inhibits mATGL but not hATGL (4, 60). They also indicate that orlistat does not show enzyme specificity and might be a pan-lipase inhibitor.

### Measuring lipoprotein lipase (LpL) activity using NBD-TAG in PC/PI vesicles

**Fig. 7:**
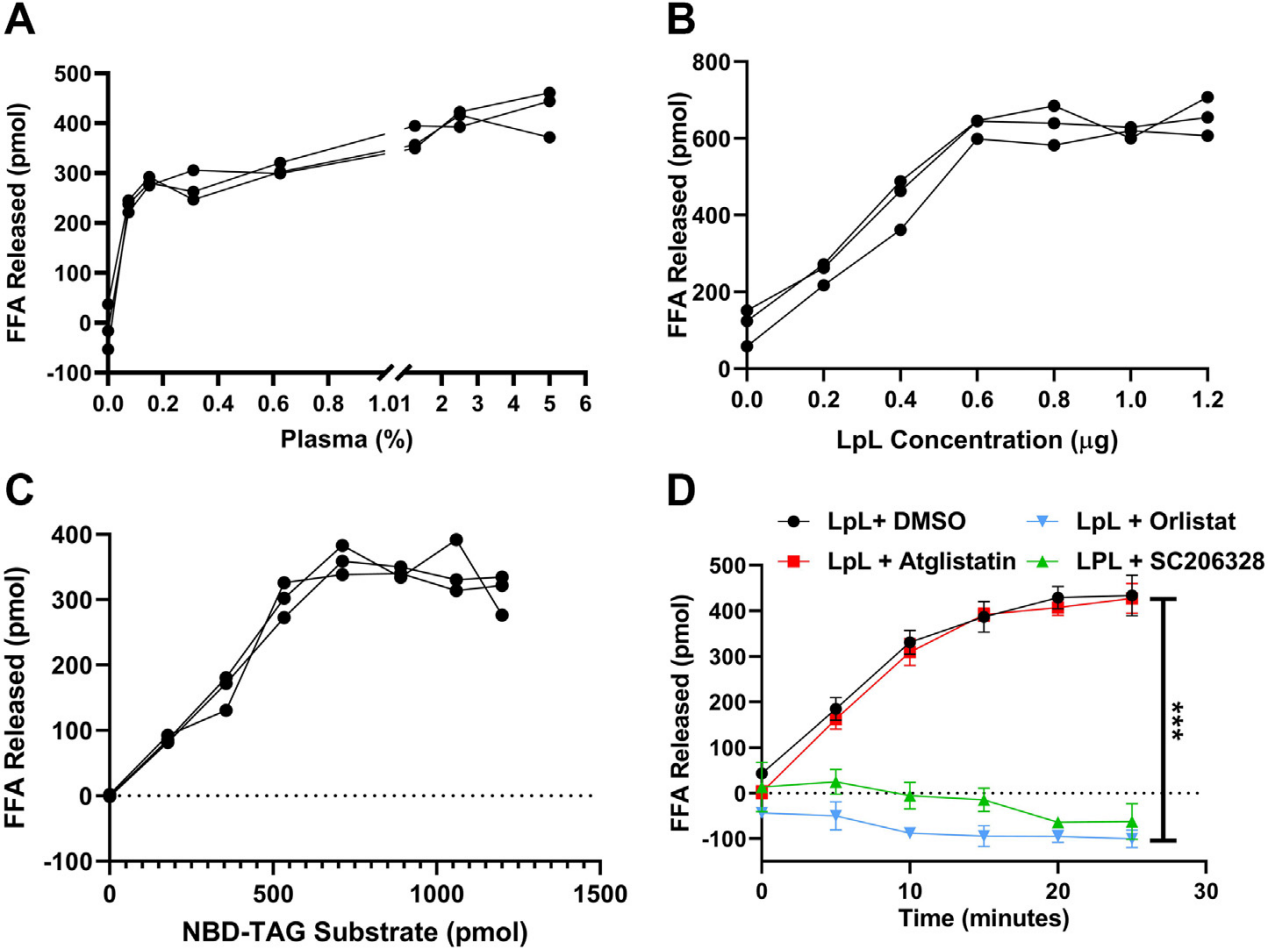
Measuring LpL activity and studying effects of different inhibitors on its activity. A: Purified bovine milk LpL (0.3 μg) was incubated with 880 pmol NBD-TAG in PC/PI vesicles with increasing amounts of mouse plasma for 15 min and FFAs released were calculated from a NBD-C6 standard curve prepared in parallel. B: Different concentrations of purified LpL were incubated with 880 pmol of substrate vesicles in the presence of 5% mouse plasma. NBD fluorescence was measured at 15 min. C: LpL (0.3 μg) was incubated with different concentrations of NBD-TAG substrate in the presence of 5% mouse plasma for 15 min. D: LpL (0.3 μg) with 5% mouse plasma was incubated with 880 pmol of NBD-TG vesicles in the presence of either DMSO, atglistatin (100 μM), orlistat (20 μM), or SC20638 (20UM). NBD fluorescence was measured at different time points. FFAs released were calculated using NBD-C6 standard curve. Error bars represent SD, n = 3; ∗∗∗*P* < 0.001, two-way ANOVA followed by multiple comparisons test.

After successfully measuring ATGL activity, we next asked whether the assay could also be used to measure the activity of other TAG lipases such as LpL, which is the major enzyme involved in lipoprotein TAG hydrolysis in the circulation (12, 13, 14). LpL is known to hydrolyze all three FAs from TAG. It first hydrolyzes FAs at sn-1 and sn-3 positions and then hydrolyzes the FAs present at sn-1 position after their migration to the sn-1 position. We used LpL purified from bovine milk as a source of enzyme and mouse plasma as a source of apoCII. First, we measured LPL activity in the presence of different amounts of plasma and found that 0.2% of mouse plasma is enough to activate LPL (Fig. 7A). Next, we studied the effect of different amounts of LpL with 5% mouse plasma (Fig. 7B). The amounts of FFAs released increased as protein concentrations increased, revealing enzyme-dependent increases in substrate hydrolysis. Based on these results, we settled on 0.3 μg LpL and 5% mouse plasma as our standard condition and then investigated the effects of substrate concentration on lipolysis (Fig. 7C). When we incubated LpL and 5% plasma with different amounts of substrate vesicles, we found clear substrate-dependent increases in the production of FFAs (Fig. 7C). We then studied the effect of different inhibitors on LpL activity and found that atglistatin had no effect, but orlistat and HSLi (SC206238) potently inhibited LpL activity (Fig. 7D). Together, these studies showed that LpL produces protein-, time-, and substrate-dependent TAG hydrolysis when incubated with NBD-TAG in PC/PI vesicles and is inhibited by orlistat and HSL inhibitor.

### Effect of increasing TAG in PC/PI emulsions on hATGL and LpL activities

**Fig. 8:**
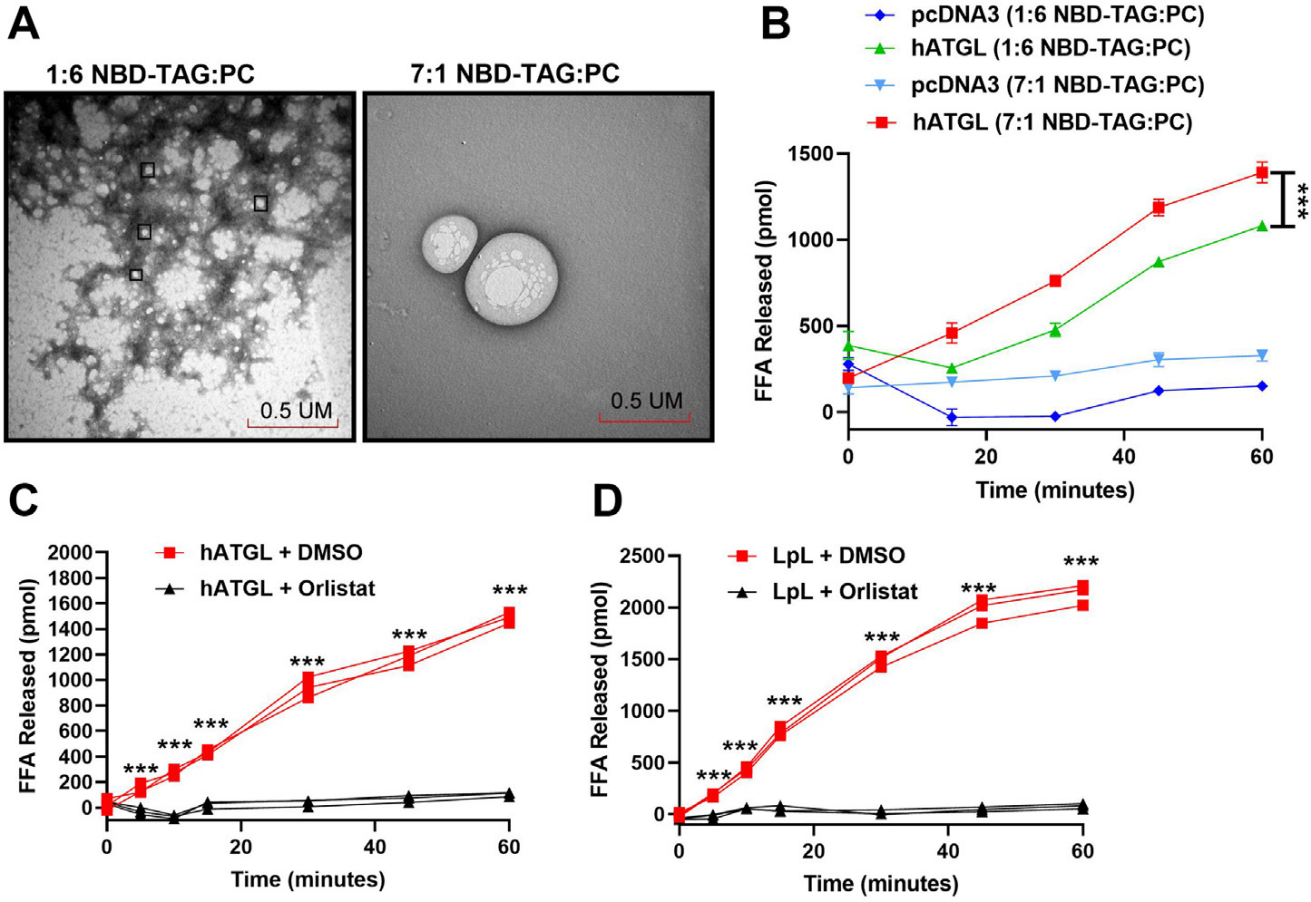
Lipase activity measurements using NBD-TAG in two different vehicles. A: TEM images of 1:6 and 7:1 NBD-TAG to PC in PC/PI emulsions at 50,000 magnification. B: pcDNA3 or hATGL transfected Cos-7 cell lysate (100 μg) were incubated with either 10 μl (880 pmol) 1:6 NBD-TAG:PC vesicles or 3.5 μl (880 pmol) 7:1 of NBD-TAG:PC in PC/PI vesicles and fluorescence was measured at different time points during the 1 h incubation. C: hATGL-transfected Cos-7 cell lysates (70 μg) were incubated with 10 μl (3,155 pmol) 7:1 NBD-TAG:PC in PC/PI emulsions in presence of either DMSO or orlistat (20 μM). Fluorescence was measured at different time points during the 1 h incubation to calculate FFAs released. D: LpL (0.3 μg) with 5% mouse plasma was incubated with 10 μl (3,155 pmol) of 7:1 NBD-TAG:PC in PC/PI emulsions in presence of either DMSO or orlistat (20 μM). NBD fluorescence was measured at different time points. FFAs released were calculated using NBD-C6 standard curve. N = 3, ∗∗∗*P* < 0.001 significance calculated by two-way ANOVA followed by multiple comparisons test.

In our assays thus far, we had used a TAG to PC ratio of 1:6. However, previously reported lipase assays used TAG emulsions with lower phospholipid concentrations (19, 25). Therefore, we synthesized new emulsions to obtain NBD-TAG to PC ratio of 7:1 while keeping the PC:PI ratio unchanged. We compared the sizes of these two types of substrate vehicles using negative staining and transmission electron microscopy (TEM) and found that the emulsions with a 7:1 NBD-TAG to PC ratio were larger (average size 244 ± 139.6 nm, n = 50) than those with a 1:6 NBD-TAG to PC ratio (average size 55 ± 11 nm, n = 50) (Fig. 8A). The new 7:1 emulsions resulted in substantial decreases in blank readings, to less than 2% of total fluorescence. Further, hATGL-transfected Cos-7 cell lysate showed significantly higher TAG hydrolysis in 7:1 NBD-TAG to PC emulsions compared with 1:6 NBD-TAG to PC emulsions (Fig. 8B). Next, we studied the effect of orlistat on ATGL and LpL using 7:1 NBD-TAG:PC in PC/PI emulsions. Orlistat was also able to inhibit enzyme activities in the 7:1 vesicles (Fig. 8C, D). Thus, these substrate emulsions are better substrates to assay ATGL and LpL activities.

### Measuring mHSL activity using 7:1 NBD-TAG:PC in PC/PI vesicles

**Fig. 9:**
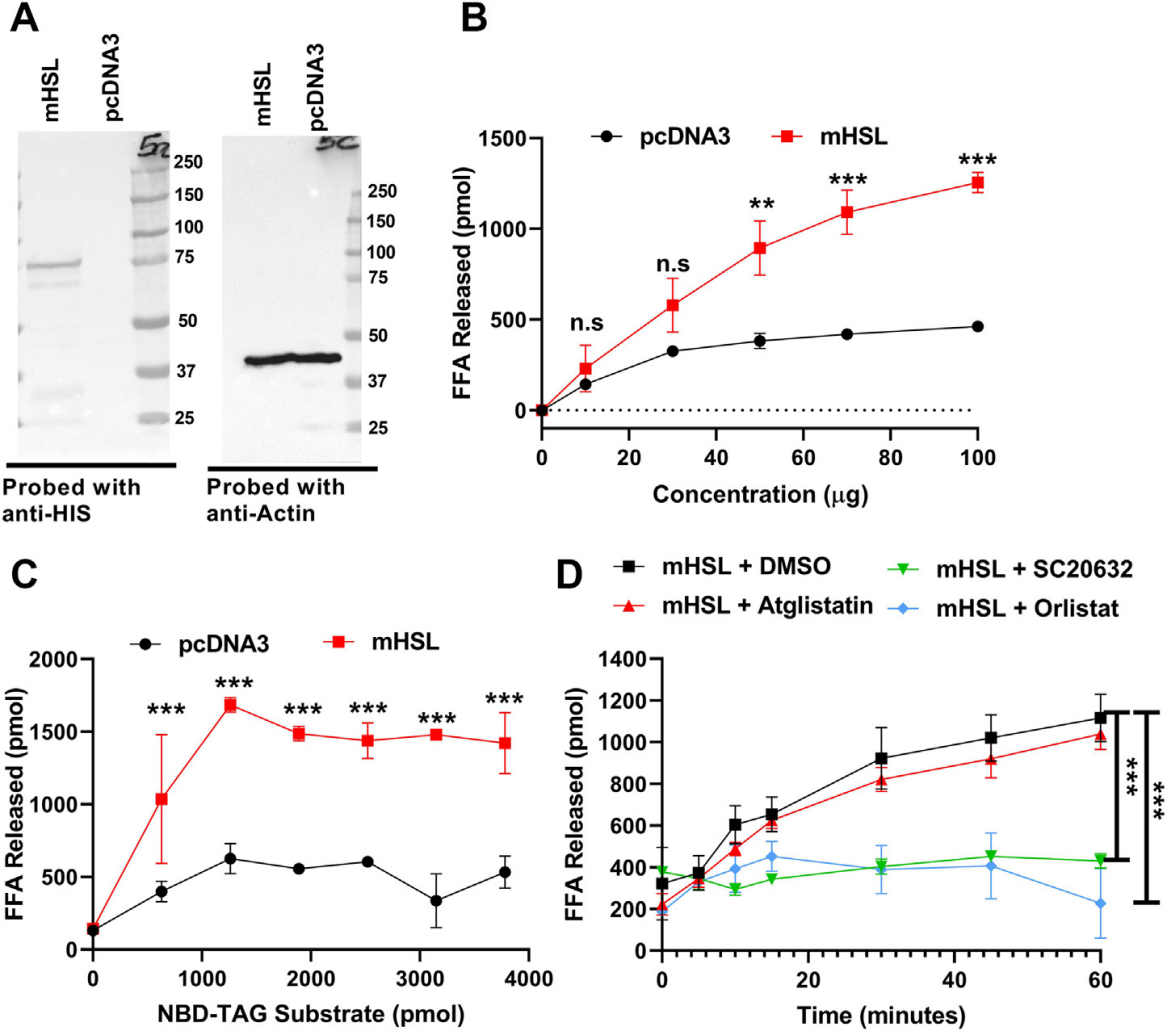
Measuring mHSL activity using 7:1 NBD-TAG:PC in PC/PI vesicles. A: Cos-7 cells were transfected with either pcDNA3 or His-tagged mHSL. Cell lysates (20 μg) were separated on a gel and probed with anti-His antibodies. In another gel, actin was probed to show equal loading of cell proteins. B: pcDNA3 or mHSL transfected Cos-7 cell lysates (100 μg) were incubated with 7:1 NBD-TAG:PC (10 μl, 3,155 pmol) substrate for 1 h. Fluorescence was measured at different time intervals during incubations to calculate FFA released. C: pcDNA3 or mHSL transfected Cos-7 cell lysates (100 μg) were incubated with different amount of 7:1 NBD-TAG:PC (10 μl, 3,155 pmol) substrate for 1 h. Fluorescence was measured at different time intervals during incubations to calculate FFA released. D: mHSL transfected Cos-7 cell lysates (100 μg) were incubated with 7:1 NBD-TAG:PC (10 μl, 3,155 pmol) substrate in the presence of either DMSO, atglistatin (100 μM), orlistat (20 μM), or SC206328 (20 μM). Fluorescence was measured at different time points during the 1 h incubation to calculate FFAs released. The graphs are representative of two to three independent experiments, each with three biological replicates. Error bars represent SD;∗∗*P* < 0.01 and ∗∗∗*P* < 0.001 as calculated by two-way ANOVA followed by multiple comparisons test.

To check whether mHSL activity can be measured using 7:1 NBD-TAG:PC in PC/PI vesicles, we transfected HIS-Tagged mHSL expression vector in Cos-7 cells (Fig. 9A). The lysates from mHSL-expressing Cos-7 cells showed significantly more FFA release in a concentration-dependent manner compared with pcDNA3-transfected Cos-7 cell lysate (Fig. 9B). Lysates from mHSL-expressing cells showed increased FFA release with increasing substrate concentrations, whereas lysates from control pcDNA3-transfected Cos-7 cells showed low hydrolysis that saturated at a lower concentration (Fig. 9C). We then studied the effect of different inhibitors on mHSL activity and found that atglistatin had no effect, but orlistat and HSLi (SC206238) potently inhibited mHSL activity (Fig. 9D). In conclusion, mHSL expressing Cos-7 cell lysate shows protein and substrate-dependent TAG hydrolysis when incubated with 7:1 NBD-TAG:PC in PC/PI vesicles and is inhibited by orlistat and HSL inhibitor. These studies demonstrate that HSL activity can be measured using 7:1 NBD-TAG:PC in PC/PI vesicles.

### Measuring pancreatic triglyceride lipase (PTL) activity using 7:1 NBD-TAG:PC in PC/PI vesicles

**Fig. 10:**
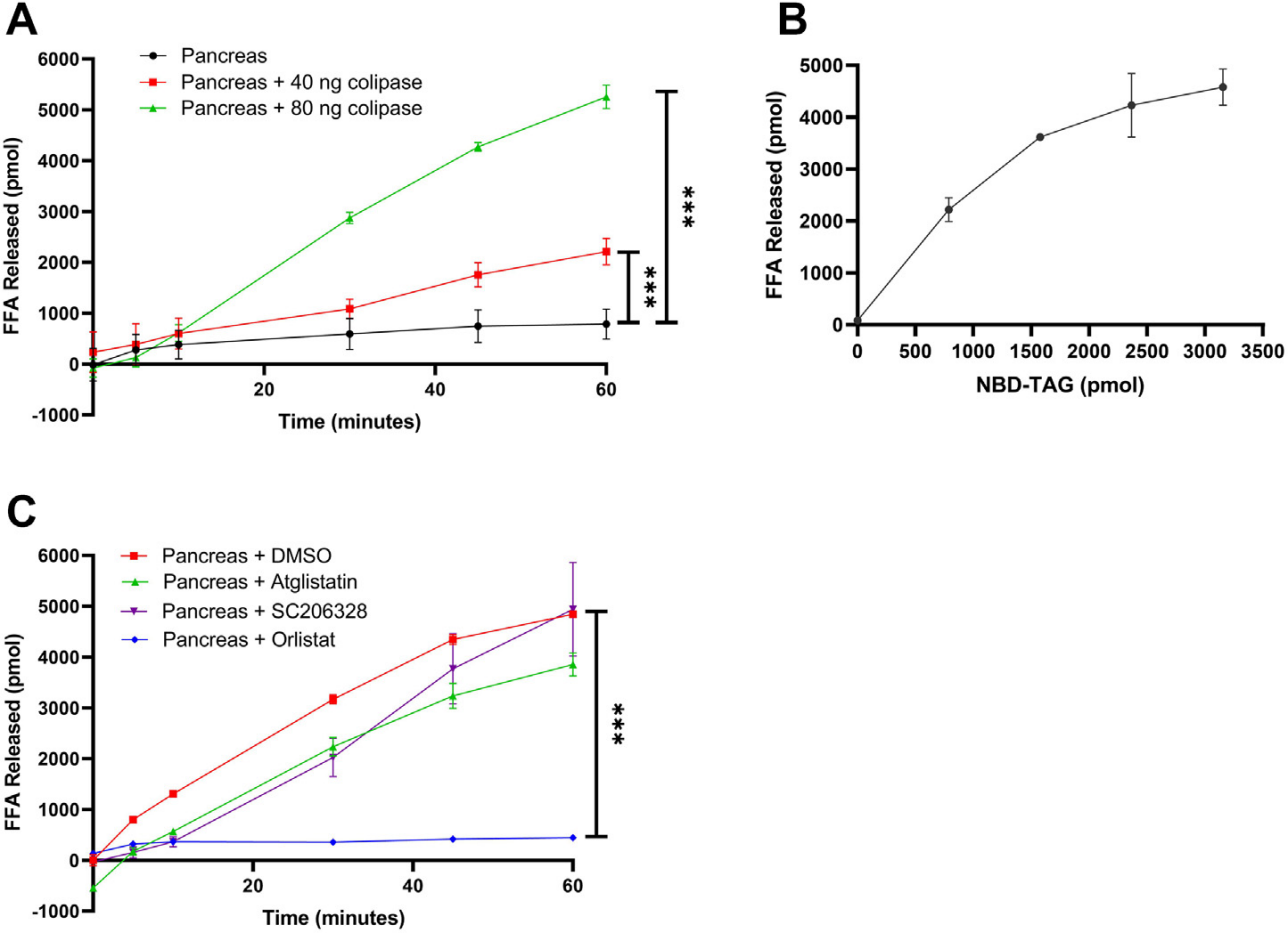
PTL activity in pancreas of fed mice and effect of different inhibitors. A: Pancreas from nonfasted C57BL/6 mice were collected and homogenized in modified buffer K. Proteins (40 μg) were mixed with NBD-TAG vesicles (10 μl, 3,155 pmol) in the absence or presence of increasing amounts of colipase. After 5 min, plates were used to measure NBD fluorescence at different time points. B: Tissue homogenates (40 μg) with 80 ng colipase were incubated with different concentrations of NBD-TAG substrate. After 1 h, plates were used to measure NBD fluorescence. C: Tissue homogenates (40 μg) with 80 ng colipase were incubated with NBD-TAG vesicles (10 μl, 3,155 pmol) in the presence of DMSO, atglistatin (100 μM), orlistat (20 μM), or SC206328 (20 μM) for 5 min. NBD fluorescence was measured at different time points. In these studies, FFA concentrations were calculated using NBD-C6 standard curve. Error bars represent SD, n = 3 technical replicates; ∗∗∗*P* < 0.001, two-way ANOVA followed by multiple comparison analysis.

To further test the applicability of the optimized assay to other TAG lipases, we next measured PTL activity in mouse pancreas. Similar to LpL, PTL fully hydrolyzes TAGs. It specifically cleaves ester bonds at sn-1 and sn-3 positions but can subsequently cleave the ester bond at sn-2 after migration to sn-1 position under alkaline conditions in the small intestine. Its catalytic activity in the intestinal lumen is enhanced by the presence of both bile acids and the cofactor colipase. Bile acids emulsify lipids, while colipase binds to lipase in 1:1 ratio, anchors the lipase to the lipid interface, and stabilizes its active conformation. In vitro, addition of calcium ions increases lipolysis most likely by reducing the surface charge of micelles (7, 9). First, we measured PTL activity in pancreatic tissue homogenates in the absence or presence of increasing amounts of colipase. We found that increasing the amount of colipase increased the amounts of FFA released (Fig. 10A), demonstrating colipase enhances FFA release by pancreatic homogenate. Next, we incubated fixed amounts of pancreatic tissue homogenates and colipase with different amounts of NBD-TAG substrate and observed a substrate-dependent increase in lipolysis (Fig. 10B). Evaluation of different inhibitors revealed that orlistat inhibits PTL activity (Fig. 10C). These studies showed that PTL exhibits a time- and substrate-dependent TAG hydrolysis when incubated with optimal concentrations of colipase and bile salts, and NBD-TAG in PC/PI vesicles, and is effectively inhibited by orlistat. Thus, the 7:1 NBD-TAG:PC in PC/PI vesicles can also serve as a substrate to assay PTL activity. These studies suggest that these substrates may be suitable to assay other lipases.

### Measuring ATGL and HSL activity in eWAT of fed and fasted mice

**Fig. 11:**
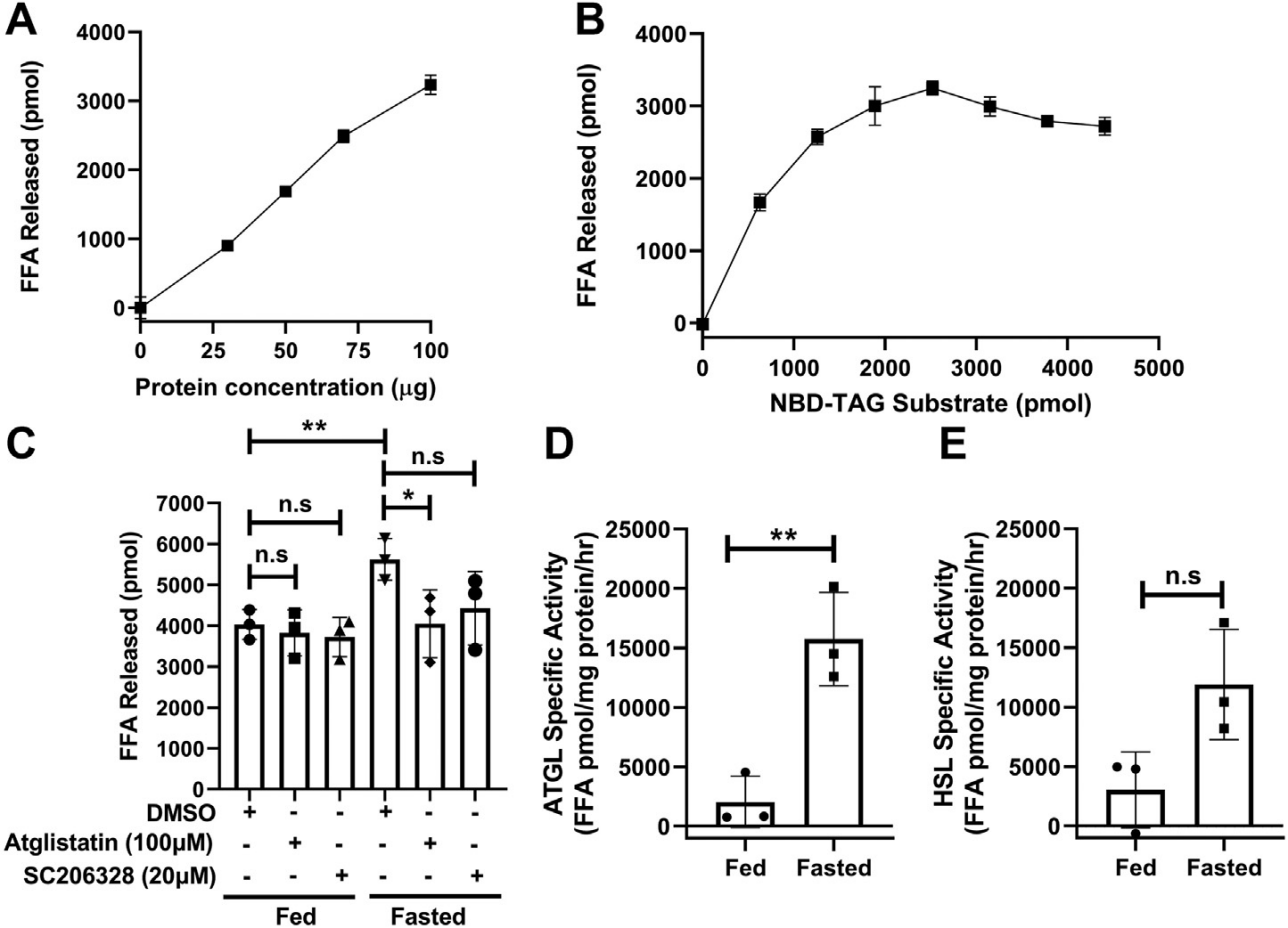
Measuring ATGL and HSL activity in eWAT of mice during fed and fasting conditions. C57BL/6 mice nonfasted (n = 3) and fasted (n = 3) overnight (16 h) were euthanized at 10 AM and eWAT was collected after transcardial perfusion with saline. A: Different amounts of eWAT homogenates from nonfasted mice were incubated with 7:1 NBD-TAG:PC vesicles (10 μl, 3,155 pmol). After 1 h, NBD fluorescence was measured. B: eWAT homogenate (100 μg) of nonfasted mice was incubated with different concentrations of NBD-TAG substrate and NBD fluorescence was measured after 1 h. C: eWAT homogenates (100 μg) from fed and fasted mice were incubated with 7:1 NBD-TAG:PC vesicles (10 μl, 3,155 pmol) in the presence of DMSO, atglistatin (100 μM), or SC206328 (20 μM). After 1 h, NBD fluorescence was measured. N = 3, error bars represent SD ∗*P* < 0.05, ∗∗*P* < 0.01 as calculated by one-way ANOVA followed by multiple comparison. D and E: The difference in FFA release in the presence and absence of atglistatin (D) or SC206328 (E) was used to calculate ATGL and HSL-specific activities, respectively, in eWAT obtained from fed and fasted mice. N = 3, error bars represent SD, significance calculated by unpaired Student's *t* test ∗∗*P* < 0.01.

We next asked whether this assay could be used to study endogenous ATGL and HSL activity in tissues. We collected epididymal white adipose tissue (eWAT) from overnight fasted C57BL/6 mice and used homogenate to study the effects of different protein and substrate concentrations. First, we incubated different amounts of eWAT homogenate with 7:1 NBD-TAG:PC vesicles and found concentration-dependent increases in FFA release (Fig. 11A). We then incubated fixed amount of eWAT tissue homogenate with different amounts of NBD-TAG substrate and observed substrate-dependent increase in FFA release until 2,520 pmol of NBD-TG; at higher concentrations, there was no further increase in FFA release indicating saturation (Fig. 11B).

Because the eWAT contains several lipases, we attempted to determine activities specific to ATGL and HSL. For this purpose, lipase assays were performed in the presence and absence of mATGL inhibitor atglistatin (100 μM). In addition, lipolysis was studied in the presence of SC206328 (20 μM), an HSL inhibitor. We found that atglistatin and SC206328 did not decrease FFA release compared with control (DMSO) eWAT homogenates obtained from fed mice. eWAT homogenate from fasted mice, however, showed significantly higher increase in FFA release compared with fed mice. Atglistatin, but not SC206328, significantly decreased FFA release in fasted mice eWAT homogenate (Fig. 11C). FFA release in the presence and absence of atglistatin was also used to calculate ATGL-specific contribution in lipolysis. We found significantly increased eWAT ATGL specific activity in fasted mice compared with fed mice (Fig. 11D). FFA release in the presence and absence of SC206328 was used to calculate HSL-specific contribution in lipolysis. We found nonsignificant increase in eWAT HSL specific activity in fasted mice compared with fed mice (Fig. 11E). Overall, these results indicate that ATGL activity in eWAT is low in fed mice, and it increases significantly during fasting.

### Recommended assay conditions using 7:1 NBD-TAG in PC/PI vesicles

We recommend incubating 7:1 NBD-TAG in PC/PI emulsions (10 μl, ∼3,155 pmol of NBD-TAG) with a source of lipase for 1 h at 37°C and measuring fluorescence every 15 min interval. Using these conditions, we found that the intra- and interassay % coefficients of variations (n = 3 replicates) were 4.3% and 15.7%, respectively. We also recommend including a standard NBD-C6 reference curve in parallel when measuring lipase activities to calculate the moles of products formed. This assay does not require extraction of FFAs. However, if they are required for further validation, they can be extracted at the end of the incubations.

## Discussion

Here, we describe a simple, rapid fluorescence-based method to determine the lipolytic activities of different lipases, particularly ATGL, HSL, LpL, and PTL because of their relevance to hyperlipidemia, obesity, and associated metabolic diseases. The assay involves incubating an enzyme source with emulsions containing NBD-TAG substrate and measuring the fluorescence of the NBD released upon hydrolysis of the substrate. The speed and simplicity make this assay suitable for high-throughput screening to identify lipase modulators that might be useful for treating metabolic diseases.

In developing this assay, we expressed hATGL in Cos-7 cells and used these cells as enzyme source. Cos-7 cells have been extensively used in lipid metabolism due to their low expression of lipid and lipoprotein metabolism genes and amenability to expressing genes via transfection (25, 61, 62). In Cos-7 cells transfected with the empty pcDNA3 vector, we detected low levels of NBD-TAG hydrolysis, suggesting the presence of low levels of lipases. Nevertheless, these lipases were too low in abundance to interfere with the measurement of ATGL activity after overexpression of the enzyme. Therefore, these cells should also be useful in developing assays for other lipases, as we have demonstrated for different lipases, after their overexpression.

A rate-limiting step in measuring lipase activities has been the need to separate products from substrates using differential solvent extraction procedures. With the use of PC/PI vesicles, we were able to eliminate this step because the increase in NBD-C6 fluorescence was several fold higher than the background fluorescence from NBD-TAG. We have previously shown that incorporation of NBD-TAG in PC vesicles quenches NBD fluorescence (47, 48). However, there remains background NBD-TAG fluorescence that is less than 10% of the total NBD-TAG incorporated into vesicles. When we used NBD-TAG packaged in PC vesicles, the increases in fluorescence due to substrate hydrolysis were small, necessitating the separation of FFA products from the NBD-TAG substrate via solvent extraction to obtain sufficiently high fluorescence to distinguish from background. Including PI in the vesicles increased NBD-TAG hydrolysis significantly without affecting background fluorescence, so that the fluorescence arising from the released NBD-C6 was significantly higher than the background from NBD-TAG. Therefore, simple subtraction of the background fluorescence from the total fluorescence was sufficient to measure enzyme activity, eliminating the need to separate FFAs from NBD-TAG. We also found that the assay could be made more sensitive by increasing the molar ratio of NBD-TAG to PC from 1:6 to 7:1. This modification substantially reduced background fluorescence, to less than 2% of the total fluorescence, and increased lipase activity substantially (Fig. 9B). These studies show that TAG to PC ratio and the inclusion of PI have significant effects on hydrolysis of NBD-TAG by lipases.

The enhancement of NBD-TAG hydrolysis by the inclusion of PI is consistent with previous studies (63). The mechanisms for this increased hydrolysis are unclear. It is likely that PI alters membrane lipid organization, allowing easier access for enzymes to its substrate. Alternatively, the acyl bonds in TAG are more facile around PI, thus perhaps making them more readily hydrolyzable by enzymes. PI is known to bind to proteins, such as cytochrome bc1 (64). Therefore, it is possible that PI facilitates the binding of lipases to substrate vehicles and increases the accessibility of neutral lipid substrates to the enzyme. More biophysical studies on membrane morphology, lipid topology, and protein access are needed to explain the effects of PI on increased NBD-TAG hydrolysis by different lipases.

We compared the efficacy of our assays with those of published assays of ATGL. Using NBD-TAG in PC vesicles, we observed specific activities of ∼2 nmol/h/mg protein. Therefore, ≥50 μg of COS-7 cell lysate overexpressing hATGL was required to measure meaningful activity. However, the specific activities were higher when NBD-TAG substrate was emulsified in PC/PI vesicles (∼10 nmol/h/mg/protein). Hence, inclusion of PI increases sensitivity by approximately 5-fold. When the TAG to PC ratio was changed from 1:6 to 7:1, the hATGL activity rose to ∼15 nmol/h/mg protein. PC/PI-stabilized radioactive triolein has been used to assay hATGL activity after its overexpression in Cos-7 cells, with a reported hATGL specific activity of ∼30 nmol/h/mg protein (19, 29). On this account, our fluorescence assay is less efficient than the use of radioactive triolein, which is expected, given that the substrate used in our assay is not a natural substrate of the enzyme. Furthermore, the concentrations and compositions of our substrate vesicles differ significantly from those used in the radioactive assay (19, 25). A further complication with this comparison is that we do not know the exact amounts of ATGL proteins present in the samples in these two different experiments performed in two different countries at two different times. Nonetheless, we found that ATGL is able to hydrolyze NBD-TAG efficiently when presented in PC/PI vesicles, especially at the higher NBD-TAG to PC ratio of 7:1.

The methods described here could be easily adapted to measure phospholipases and cholesterol ester hydrolases, depending on the availability of different NBD-labeled substrates. If successfully established, all of these assays will be suitable for high-throughput screening to discover modulators that affect different enzyme activities. A possible issue that could arise in developing phospholipase and cholesterol esterase assays is high background fluorescence due to inadequate quenching of the fluorophore. This can potentially be mitigated by varying phospholipid composition. If such problems persist, they can be circumvented by adding back the extra step of extracting FFAs released into aqueous phase and measuring the fluorescence.

This assay does present some drawbacks. First, NBD-TAG contains C18:1 fatty acids at the sn-1 and sn-3 positions of the glycerol backbone. At the sn-2 position, however, a medium-chain-length fatty acid (C6), linked with the fluorophore NBD, is esterified to the hydroxyl group. Thus, the NBD-TAG is not a physiologic substrate. Although smaller than other fluorophores, NBD is still bulky and may thus alter or hinder the access of enzymes to the substrate. Second, studies of a specific lipase depend on the availability of purified enzyme or expression plasmids for individual enzymes. The assay can be made specific if potent-specific inhibitors are available; however, our analysis of commercially available inhibitors suggests that they are not very specific. The assays described here can be used to identify more specific inhibitors. Despite these drawbacks, these fluorescence-based assays are of interest as they forgo the use of radioactive substances.

As an alternative, radiolabeled substrates have great advantage because the label does not differ from the natural substrate, providing the best way to measure enzyme activities. However, the use of such substrates requires special training, handling, and disposal of radioisotopes. More importantly, in the case of lipases, these assays require separating substrates from products. These assays are also not suitable for measuring real-time enzyme kinetics as they have to be terminated at a given time to obtain information about time-dependent kinetics. Moreover, radiolabel-based assays are difficult to optimize for high-throughput screening. Therefore, nonradioactive assays remain of great interest, and the new assay we describe here shows promise in overcoming some of these drawbacks.

Since lipases are increasingly being used for various commercial purposes, this fluorescence-based method could be valuable for automated analyses of lipase activities and for high-throughput screening to identify specific activators and inhibitors of different enzymes. Furthermore, recent publications provide proof of concept that chemical inhibition of ATGL might be a good therapeutic strategy for treating obesity and related metabolic diseases as mice treated with atglistatin showed reduced adipose tissue lipolysis, weight gain, and insulin resistance (60, 65, 66, 67). Atglistatin is not a potent inhibitor of hATGL (4, 60); the improved assay described could be useful for identifying a more potent hATGL inhibitor via high-throughput screening. Similarly, there has been a recent focus on the possibility of enhancing intravascular lipolysis of lipoproteins by inhibiting LpL inhibitors, such as apoCIII (68) and ANGPTL3 (69), to reduce plasma lipid levels. Inhibition of natural inhibitors of LpL has been successfully exploited to lower plasma lipids. This assay could facilitate identification of small molecules that could enhance LpL activity by directly modulating the activity of the enzyme, its activator apoCII, or inhibitors. Meanwhile, orlistat is the only clinically approved drug to treat and manage obesity, warranting an ongoing search for safe and effective alternatives (70). This assay could be used to evaluate potential candidate inhibitors of PTL activity.

In conclusion, here we report a simple, rapid fluorescence assay utilizing NBD-TAG as substrate for measuring lipolytic activity. The assay's ability to measure inhibitory effects and the ease of conduct on a large scale make it an excellent platform for automation and for screening compounds for antilipolytic activity against any given lipase. It can also be used to identify activators that enhance activities of different lipases as we have demonstrated for LpL and PTL. As this assay is fluorescence-based, it is relatively environmentally friendly, forestalling the use of radioactivity, at the same time promotes a safer research workplace.

## Data availability

All the data is included in the manuscript.

## Conflict of interest

The authors declare that they have no conflicts of interest with the contents of this article.
